# Indocyanine green-assisted chyle leak identification and ligation after left carotid-subclavian bypass: a case report

**DOI:** 10.1093/jscr/rjag192

**Published:** 2026-03-26

**Authors:** Diego Soto, Ricardo Jiménez, Luis Contreras, Valentina San Martín, Paulo Cassis, Sebastián Morales, Sebastián González, Gabriel Seguel

**Affiliations:** Vascular Surgery Unit, Hospital Dr. Sótero del Río, Santiago, Chile; Vascular Surgery Department, Pontificia Universidad Católica de Chile, Santiago, Chile; Surgery Department, Pontificia Universidad Católica de Chile, Santiago, Chile; Interventional Radiology Unit, Hospital Dr. Hospital Dr. Sótero del Río, Santiago, Chile; Vascular Surgery Unit, Hospital Dr. Sótero del Río, Santiago, Chile; Vascular Surgery Unit, Hospital Dr. Sótero del Río, Santiago, Chile; Vascular Surgery Unit, Hospital Dr. Sótero del Río, Santiago, Chile; Vascular Surgery Unit, Hospital Dr. Sótero del Río, Santiago, Chile

**Keywords:** chyle, indocyanine green, fluorescence, carotid-subclavian bypass, vascular grafting

## Abstract

Cervical chyle leak (CL) is a rare but potentially serious complication following carotid–subclavian bypass (CSB) during cervical debranching of supraaortic vessels. We report the case of a 62-year-old male who developed a postoperative CL after staged CSB and thoracic endovascular aortic repair. Initial conservative management with dietary modification and octreotide resulted in temporary improvement; however, recurrence occurred after reintroduction of dietary fats. Surgical re-exploration with intraoperative indocyanine green (ICG) fluorescence imaging enabled precise localization and successful ligation of the leaking lymphatic vessel. Postoperative recovery was uneventful, with complete resolution of the CL. This case highlights the value of ICG-assisted imaging as a safe and effective adjunct for the diagnosis and surgical management of refractory cervical CL, particularly in patients with prosthetic vascular grafts.

## Introduction

Chyle leak (CL) after head and neck surgery is an infrequent but known complication, which presents in up to 2%–8% of neck dissections [1]. Due to the variable anatomy and fragile tissue of the thoracic duct (TD) [2], the inadvertent iatrogenic lesion is the most common cause of postoperative cervical CL [1, 3, 4].

Cervical debranching of supraaortic vessels (CDSAV), especially the left carotid to left subclavian artery (LSA) bypass (CSB) is a wide known revascularization strategy to preserve patency of the LSA with low morbidity [4], either for occlusive disease or achieving a safe proximal landing zone during thoracic endovascular aortic repair (TEVAR) that involves the distal aortic arch [5].

Cervical CL is a rare complication after CSB [6] that requires a prompt diagnosis to achieve its resolution, where the assisted identification with Indocyanine green (ICG) can be useful [7].

## Case presentation

**Figure 1:**
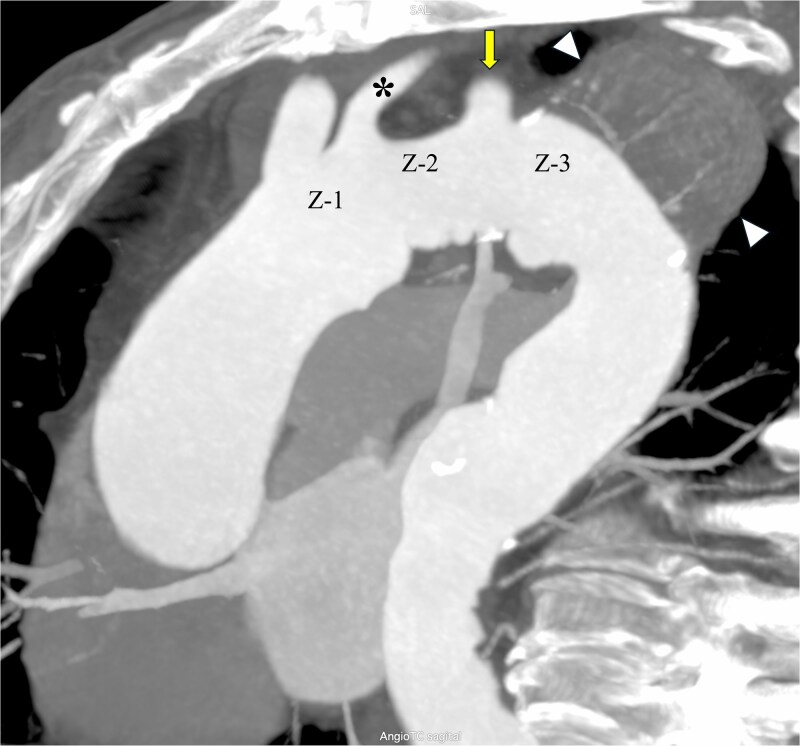
Preoperative computerized tomography angiography of the aortic arch. The saccular aneurysm in the distal arch (zone 3; Z-3) is depicted (arrowheads) and its close anatomical situation with the left subclavian artery (arrow). Therefore, a safe proximal landing zone for endograft deployment in zone 2 (distal to the left common carotid artery [asterisk]; Z-2) was planned, performing a left carotid-left subclavian artery bypass using a polytetrafluoroethylene (PTFe) graft.

62-year-old male with hypertension, heart failure (left ventricular ejection fraction [LVEF] 40%), pulmonary hypertension and a pulmonary embolism with anticoagulation treatment, was on follow-up for presenting a 6.1 cm saccular aneurysm of the distal aortic arch (Zone 3) (Fig. 1). Due to the reduced LVEF, an open thoracic aortic repair was discarded and -due to the absence of a secure length of proximal landing zone distal to the LSA- a CDSAV of the LSA with a CSB and TEVAR in Zone 2 (distal to the left common carotid artery) was scheduled.

**Figure 2:**
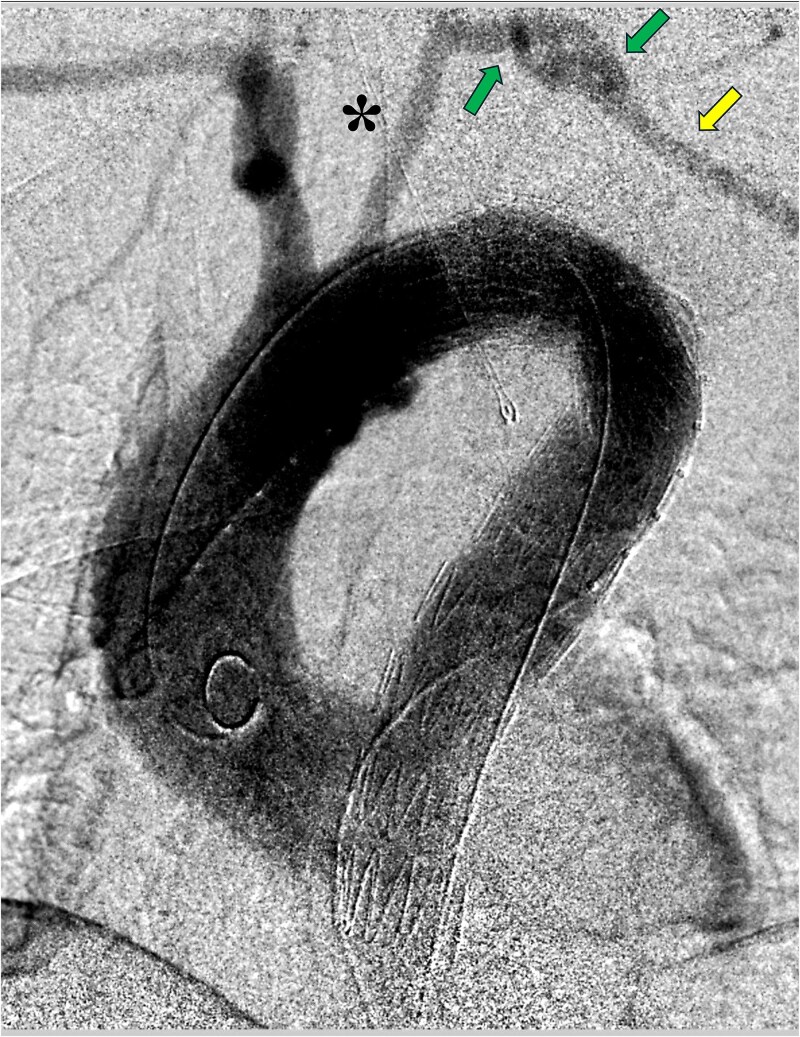
Intraoperative aortography after performing TEVAR in zone 2 (distal to the left common carotid artery [asterisk]) of the aortic arch (Zenith Alpha™, Cook Medical©). The left subclavian artery (rightarrow) is perfused through the carotid-subclavian bypass (left and central arrows).

**Figure 3:**
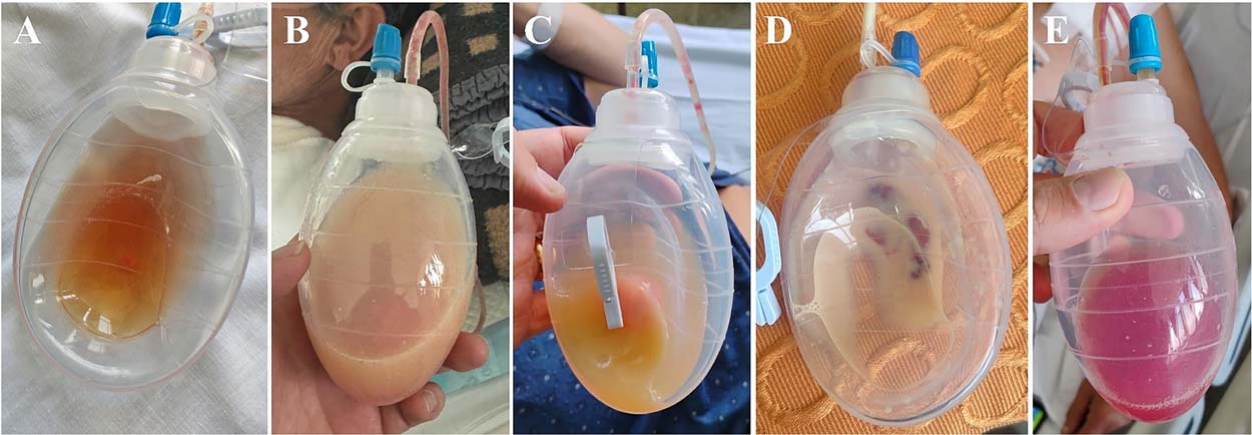
Evolution and aspect of the drainage content throughout the hospitalization. During the first days after the carotid subclavian bypass and TEVAR, a citrine serohematic fluid was identified in the drainage collector (A). Then, 24 hrs after reinitiating oral intake, the drainage turned to a milkier aspect with an increase in the daily output (B). Once confirmed the chylous etiology (after the fluid analysis) dietary fat restrictions and octeotride were initiated. The drainage fluid recovered its previous citrine aspect associated with a daily output decrease (C). Due to the initial response to conservative measures, the oral intake of fat was reintroduced and the fluid drainage turned again into a milky dense fluid (D). Due to this, conservative measures were considered insufficient and surgical reexploration with indocyanine-green assistance was performed. After the surgical ligation of the chyle-leak, the drainage fluid aspect turned serohematic with low output within the first postoperative 24 hrs (E). The drainage was retrieved 48 hrs post chyle-leak ligation, and the patient was discharged.

The CSB was performed without incidents using an 8 mm ringed prosthetic vascular graft and ligation of the LSA (proximal to the subclavian anastomosis), leaving an aspirative drainage. In a second stage (48 hrs later) TEVAR was performed without incidents (Fig. 2). During this surgical interval, the drainage presented an output of 360 ml/day of serohematic appearance. On postoperative day 4 after CSB, and after reinitiating a normal diet, the appearance of the drainage content turned into a milky fluid increasing its output to 500 ml/day (Fig. 3). The analysis of the fluid was positive for triglycerides (2238 mg/dl), confirming the presence of a CL.

Dietary measures (non-fat and high protein intake) were initiated, associated with subcutaneous octeotrid administration (100 μg/q8h) [1]. The output decreased to 100 ml/day, returning to a serohematic aspect for 5 days. Then, a medium-fatty acid diet was introduced (maintaining octeotride), and after 24 hrs, the output of the drainage increased to 500 ml/day with a milkier appearance. Due to this, the conservative management was considered unsuccessful, and given the presence of a prosthetic graft, we decided to perform a surgical exploration and ligation of the CL using ICG assistance.

**Figure 4:**
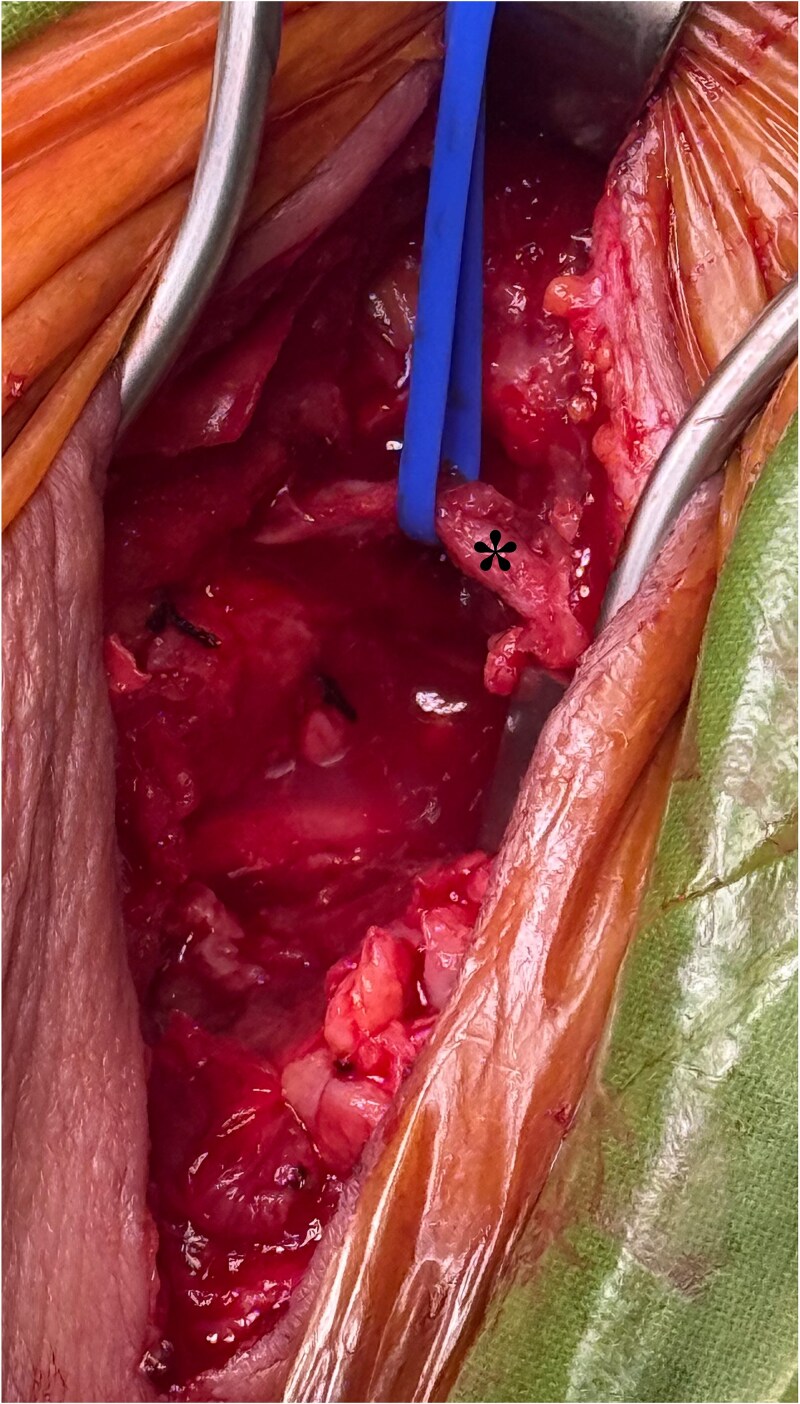
Reexploration of the cervical incision and surgical site. After the cervical fascia was reopened, the surgical bed was exposed, where it was identified a moderate content of the chyle fluid. The left yugular vein was controlled with vascular loops (asterisk) for anatomical reference. Two silk sutures are identified from the initial carotid-subclavian bypass surgery. After aspiration of the chyle fluid and tissue debridement, the vascular graft of the bypass was evidenced to be in contact with the chyle.

**Figure 5:**
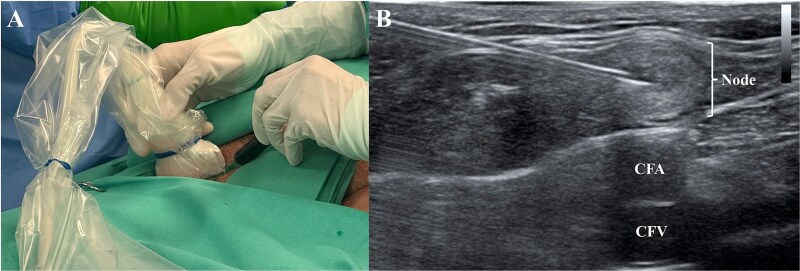
At the same time the cervical reexploration was being performed, the right inguinal area was prepared and under ultrasonographic guidance (A) using a 21G needle, an inguinal lymph node (B) was punctured and the indocyanine green was administered (0.2 mg/kg). CFA: Common femoral artery; CFV: Common femoral vein.

**Figure 6:**
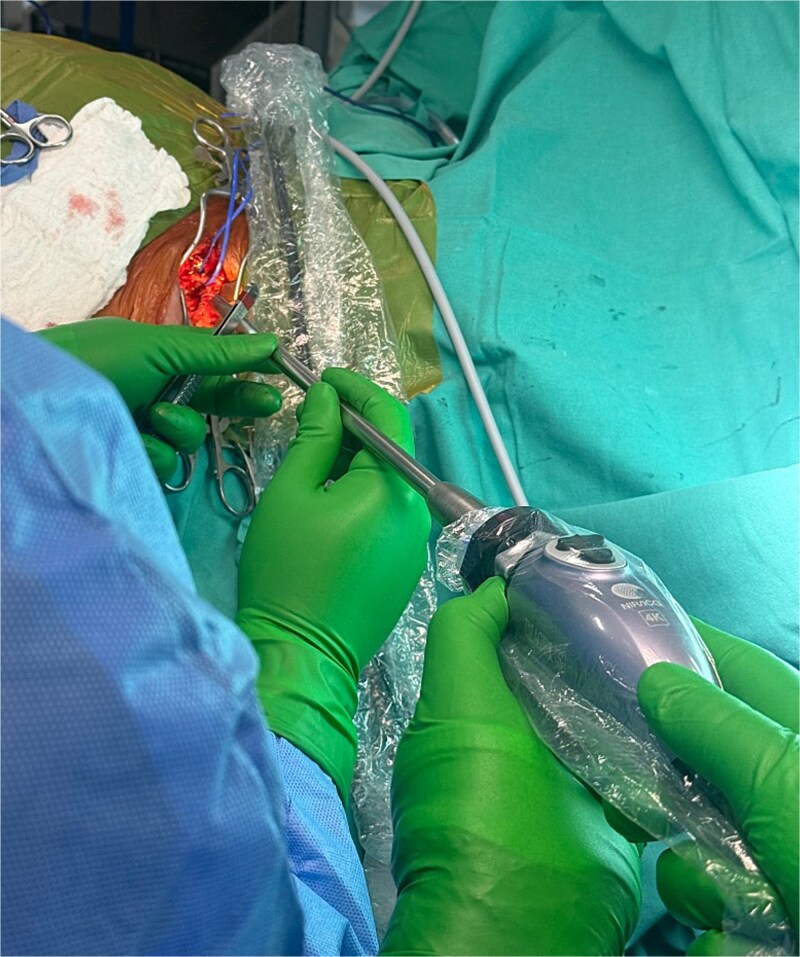
Forty minutes after the indocyanine green administration, the Hopkins® 0° NIR/ICG optic camera was directly situated and pointed into the surgical field.

**Figure 7:**
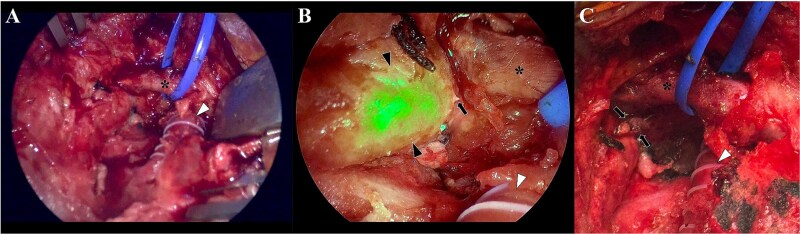
Camera view of the surgical field, without the near-red feature (A), where is depicted the bypass (arrowhead) and the left jugular vein (asterisk). Two visible silk sutures from the previous intervention are also identified. With the near-red feature (B), the thoracic conduct is clearly visualized due to the presence of the indocyanine-green (black arrowheads). This allowed to precisely identify that the leak came from thoracic duct (black arrow), in an anatomical position near the bypass (white arrowhead) and the left jugular vein (asterisk). Once the leak was identified, a clip ligation was performed with a metallic clip (C; black arrows) and the incision was closed in a standard fashion.

Under general anesthesia, re-exploration of the cervical site was carried out (Fig. 4). At the same time, under ultrasound guidance [8], a right inguinal lymphnode [8] was punctured with a 21G needle (Fig. 5) and the ICG was administered (0.2 mg/kg, 15 mg total) [9]. After 40 minutes, using a near-infrared camera (Hopkins® 0° NIR/ICG Optic, Storz. Tuttlingen, Germany; Fig. 6), a green bright structure was visualized. Direct and precise exploration of the lymphatic structure was carried out and the CL was identified and ligated (Fig. 7). After confirming no other CLs, the wound was closed leaving an aspirative drainage.

During the first 24 hrs the drainage output decreased to <50 ml/day with a serohematic appearance (Fig. 6). After 48 hrs, a non-fat diet was resumed, with no changes in the drainage’s aspect or output, being withdrawn at the 3rd postoperative day after the CL ligation. The patient was discharged without evidence of the CL at the 4th postoperative day (18 days total). Octeotride administration was maintained for another week after discharge. At 1 month follow up the patient is asymptomatic, without surgical site complications, returning to a normal dietary intake.

## Discussion

Cervical CL after CSB is a rare (0.7%) [7] but potentially serious complications of CDSAV for hybrid aortic arch surgery. Their occurrence is mostly attributable to the fragile structure, transparency and size reduction (due to the preoperative fasting period) and a marked anatomical variability of the TD -particularly near its cervical termination at the left subclavian vein [2]- where precise identification of lymphatic anatomy is often challenging, increasing the risk of inadvertent injury.

Prompt diagnosis is critical to properly initiate treatment. Conservative measures (dietary fat restrictions; total parenteral nutrition and octeotride) [1, 3] are usually effective, but in some cases, persistent CL increases the in-hospital stay and may developed malnutrition and lymphopenia, increasing the risk of local and systemic infections [4]. Therefore, when conservative measures are insufficient, surgical treatment should be considered.

Fluorescence imaging with ICG has emerged as a valuable adjunct for both the prevention and management of CLs [8]. Following peripheral or nodal injection [9–11], ICG binds to plasma proteins and is transported through the lymphatic system [12], allowing real-time visualization of the TD and its tributaries under near-infrared imaging. This technique overcomes many limitations of white-light visualization, particularly in reoperative fields, obese patients, or anatomically distorted surgical planes.

Studies have demonstrated that ICG fluorescence significantly improves TD identification rates and facilitates targeted ligation of CLs [13]. By enabling clear delineation of lymphatic flow, ICG allows localizing more precisely the site of leakage and confirming successful control intraoperatively, reducing the risk of persistent or recurrent CL. This is especially relevant in vascular procedures involving prosthetic grafts, where ongoing lymphatic leakage increases the risk of graft infection and wound complications [7]. ICG also may be used as a preventive tool, where prophylactic visualization of the TD during CDSAV may allow to avoid injury altogether or perform preemptive ligation when necessary. ICG fluorescence imaging is associated with a favorable safety profile [12], low additional operative time, and low-cost relative to the morbidity of untreated CL. Its integration into surgical workflows does not require specialized infrastructure beyond near-infrared imaging systems, which are widely available for other surgical specialties.

## Conclusion

Cervical CL after CSB is a rare but potentially severe complication, particularly when prosthetic vascular grafts are involved. The use of ICG it’s a safe and useful aid in cases when conservative measures have failed, providing precision for the CL’s identification, especially in reoperative fields when conservative measures fail.

## References

[1] Delaney SW, Shi H, Shokrani A et al. Management of chyle leak after head and neck surgery: review of current treatment strategies. Int J Otolaryngol 2017;2017:8362874. 10.1155/2017/8362874 28203252 PMC5288539

[2] Ratnayake CBB, Escott ABJ, Phillips ARJ et al. The anatomy and physiology of the terminal thoracic duct and ostial valve in health and disease: potential implications for intervention. J Anat 2018;233:1–14. 10.1111/joa.12811 29635686 PMC5987815

[3] Leović D, Pastorčić Grgić M, Gugić Radojković I et al. Management of chyle leak following head and neck surgery: review of current treatment strategies and algorithmic approach to the treatment. Acta Clin Croat 2022;61:88–95. 10.20471/acc.2022.61.s4.11 PMC10218081 37250658

[4] Sriram K, Meguid RA, Meguid MM. Nutritional support in adults with chyle leaks. Nutrition 2016;32:281–6. 10.1016/j.nut.2015.08.002 26472113

[5] Voigt SL, Bishawi M, Ranney D et al. Outcomes of carotid-subclavian bypass performed in the setting of thoracic endovascular aortic repair. J Vasc Surg 2019;69:701–9. 10.1016/j.jvs.2018.07.022 30528402

[6] Konstantinou N, Debus ES, Vermeulen CFW et al. Cervical debranching in the endovascular era: a single centre experience. Eur J Vasc Endovasc Surg 2019;58:34–40. 10.1016/j.ejvs.2018.12.010 31204185

[7] Kucera J, Lee KF, Koelling E et al. A protocolized approach to chyle leaks to mitigate prosthetic graft infection. J Vasc Surg Cases Innov Tech 2025;11:101808. 10.1016/j.jvscit.2025.101808 40488187 PMC12141895

[8] Evans MJ, Bunola-Hadfield EE, Sowkoor JS et al. The efficacy of indocyanine green fluorescence in facilitating thoracic duct visualisation and mitigating injury in cervicothoracic surgery: a systematic review and meta-analysis. Br J Oral Maxillofac Surg 2025;63:349–56. 10.1016/j.bjoms.2025.03.013 40374490

[9] Narushima M, Yamamoto T, Ogata F et al. Indocyanine green lymphography findings in limb lymphedema. J Reconstr Microsurg 2016;32:072–9. 10.1055/s-0035-1564608 26422172

[10] Santoro A, Soto D, Viscardi S et al. Robotic-assisted thoracoscopic thoracic duct ligation with fluorescence imaging with indocyanine green for chylothorax after thoracoabdominal aneurysm repair. J Vasc Surg Cases Innov Tech 2025;11:101807. 10.1016/j.jvscit.2025.101807 40496662 PMC12151224

[11] Bassi M, Vannucci J, Venuta F et al. Effectiveness of indocyanine green fluorescence for the identification of thoracic duct in recurrent idiopathic chylothorax. Interact Cardiovasc Thorac Surg 2020;31:284. 10.1093/icvts/ivaa080 32706031

[12] Mytych W, Bartusik-Aebisher D, Aebisher D. The medical basis for the photoluminescence of indocyanine green. Molecules 2025;30:888. 10.3390/molecules30040888 40005197 PMC11858079

[13] Chakedis J, Shirley LA, Terando AM et al. Identification of the thoracic duct using indocyanine green during cervical lymphadenectomy. Ann Surg Oncol 2018;25:3711–7. 10.1245/s10434-018-6690-4 30076554 PMC6181776

